# Investigating a Lock-In Thermal Imaging Setup for the Detection and Characterization of Magnetic Nanoparticles

**DOI:** 10.3390/nano10091665

**Published:** 2020-08-25

**Authors:** Lukas Steinmetz, Christoph Kirsch, Christoph Geers, Alke Petri-Fink, Mathias Bonmarin

**Affiliations:** 1 Adolphe Merkle Institute, University of Fribourg, 1700 Fribourg, Switzerland; lukas.steinmetz@unifr.ch (L.S.); christoph.geers@unifr.ch (C.G.); alke.fink@unifr.ch (A.P.-F.); 2 Institute of Computational Physics, School of Engineering, Zurich University of Applied Sciences, 8400 Winterthur, Switzerland; christoph.kirsch@zhaw.ch

**Keywords:** measurement instrument, thermal imaging, magnetic nanoparticles, lock-in thermal imaging

## Abstract

Magnetic hyperthermia treatments utilize the heat generated by magnetic nanoparticles stimulated by an alternating magnetic field. Therefore, analytical methods are required to precisely characterize the dissipated thermal energy and to evaluate potential amplifying or diminishing factors in order to ensure optimal treatment conditions. Here, we present a lock-in thermal imaging setup specifically designed to thermally measure magnetic nanoparticles and we investigate theoretically how the various experimental parameters may influence the measurement. We compare two detection methods and highlight how an affordable microbolometer can achieve identical sensitivity with respect to a thermal camera-based system by adapting the measurement time. Furthermore, a numerical model is used to demonstrate the optimal stimulation frequency, the degree of nanomaterial heating power, preferential sample holder dimensions and the extent of heat losses to the environment. Using this model, we also revisit some technical assumptions and experimental results that previous studies have stated and suggest an optimal experimental configuration.

## 1. Introduction

Superparamagnetic iron oxide nanoparticles (SPIONs) have become a topic of interest for biomedical applications due to their capability to generate heat upon stimulation with an external alternating magnetic field (AMF) [1,2,3]. These nanoparticles (NPs) are well known for their bio-compatibility, which can be further enhanced by surface functionalization, rendering them non-toxic for the human body and thereby making them ideal candidates for magnetic hyperthermia or magnetic resonance imaging (MRI) [4,5]. Several synthesis routes for SPIONs have been published over the last years, allowing decisive control over NP size and shape in order to be precisely tailored to address the final NP application [6,7,8,9,10,11].

Alongside the scientific interest in SPIONs, their usage in magnetic fluid hyperthermia (MFH) has been established [2,5,12]. Hyperthermia treatments are used in medical cancer therapy with the goal to elevate the temperature of the cancer tissue to a critical threshold, which ultimately results in the death of tumor cells (i.e., apoptosis) [13,14]. While different application settings exist, such as whole-body hyperthermia, only MFH involves the use of SPIONs. For a successful therapy the NPs have to be applied in close proximity to the cancer site and subsequently be stimulated by an AMF [15].

The control over the NP heat generation is crucial as apoptosis is desired only for the cancerous cells and therefore the temperature elevation has to be restricted to the malignant tissue. On the other side, if the heat generation does not reach the critical threshold, cell death will not be induced [5]. Naturally, various parameters, such as the magnetic field strength and frequency, but also SPION concentration, stability, size, shape and surface functionalization, strongly influence the heating power [2,12,16]. Hence, these conditions have to be properly tested to maximize the therapeutic efficiency. In clinical environments, AMFs are usually operating at a frequency of 100 kHz and at magnetic field strengths of 2 to 15 kA/m [17]. As a broad range of SPIONs have been fabricated in the recent years analytical characterization methods need to be able to precisely quantify the generated heat under varying environments. While basic material analysis and testing in research labs is usually performed in water, SPIONs are exposed to varying pH values, viscosities and biochemical milieus in the cellular environment [3].

Therefore, several calorimetric and magnetic measurement methods have been established for the analysis of SPIONs heating capabilities in an AMF, which most commonly include the usage of fiber optics, pyrometers, or thermal cameras [18,19]. These techniques determine the heating slope β in K/s that when multiplied by the sample heat capacity *C* and divided by the mass of the magnetic material $m_{MNP}$ gives the specific absorption rate (SAR) in W/g, which is used in the literature to experimentally quantify the heating efficiency of SPIONs for a specific AMF intensity [15]

(1) $$\mathrm{SAR}=\frac{\beta C}{m_{MNP}}.$$

Recently, lock-in thermal imaging (LIT) was reported as a suitable analysis tool for the characterization of SPIONs in an AMF [20,21,22]. LIT is based on a periodic thermal stimulation at a defined frequency $f_{0}$, referred to as stimulation frequency in this study. As a thermal imaging method, LIT enables the simultaneous investigation of several samples (i.e., several cuvettes containing liquid samples) and is therefore time-efficient. It can also reveal potential inhomogeneities in a solid sample (i.e., SPIONs incorporated in a gel or tissue). In LIT, the signal-to-noise ratio is significantly improved compared to other active thermal imaging techniques due to the averaging properties of the method and its ability to reject slowly varying thermal artifacts coming from the environment [23]. If an experiment can be mathematically modeled, LIT has the ability to deliver quantitative and semi-quantitative information on the NP heating slope [20,21,22,24]. The method is capable of resolving temperature differences, originating from magnetic NPs, which differ only slightly in size or shape and is highly sensitive to NP concentration. Additionally, the impact of surface functionalization on the NP heating capabilities can be investigated [25]. Another advantage of lock-in thermal imaging lies in its flexibility regarding the stimulation source. While SPIONs are commonly stimulated by an AMF to induce relaxation processes and consequently generate heat, light can be used to irradiate a broad range of NP systems, e.g., plasmonic gold (Au) or silver (Ag) NPs [26]. In this context LIT was used to investigate the heat generation of plasmonic NP systems and polydopamine NPs, which are both potential candidates for photothermal treatments [24,27]. While LIT is by far not the only method that can be applied for the analysis of SPIONs heat generation for magnetic hyperthermia treatments, it exhibits distinct advantages over other common techniques, such as standard thermography (ST), fibre optic cables (FOC) or thermocouples (TC), as highlighted in Table 1.

In this work, we describe in detail the LIT measurement apparatus developed to specifically investigate temperature changes in liquid samples containing magnetic NPs such as SPIONs, and we evaluate its performance using numerical modeling and experimental results from previous studies. The preceding sample holder design [22] as well as the mathematical method employed to retrieve quantitative information from the experimental data are explored in depth and re-visited. Furthermore, to facilitate the translation of LIT as research tool to industry, an affordable setup is proposed and experimentally validated.

## 2. Materials and Methods

### 2.1. Experimental Setup

In this subsection we present the setup specifically designed to investigate temperature changes in liquid samples containing magnetic NPs in a laboratory setting, as well as an affordable version using a microbolometer camera more suitable for industry.

**Figure 1:**
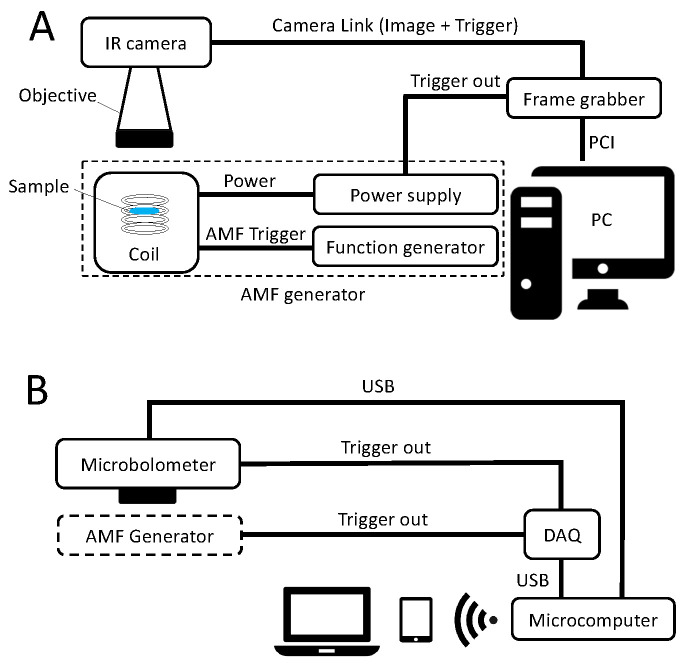
Measurement apparatus designed to investigate thermal signals of magnetic NPs in an AMF. (**A**): Schematic diagram of the laboratory setup. The alternating magnetic field is generated using a commercial coil system (MagnethermTM V1.5, nanoTherics, Warrington, UK). The frame grabber card transfers the temperature images in real time to a personal computer for further processing, and it synchronizes the stimulation. (**B**): Schematic description of a potential alternative affordable setup. The temperature images acquired by a microbolometer camera are sent in real time via USB to a microcomputer for processing without the need of an additional frame grabber. A web server running on the microcomputer allows for a wireless data transfer to a laptop or tablet. As the camera is operating on a rolling frame basis it acts as a master clock sending trigger signals to the stimulation source.

Part A of Figure 1 illustrates the laboratory instrument. The AMF is generated using a coil system (MagnethermTM V1.5, nanoTherics, Warrington, UK) that comprises a function generator (SFG-2004, GW Instek, New Taipei City, Taiwan), a water-cooled coil and a laboratory power supply (EA-PS 3032-20B, EA Elektro Automatic, Viersen, Germany). The output of the AMF is a sine wave which frequency and amplitude can be adjusted. The thermal camera (Onca-MWIR-InSb-320, XenICs, Leuven, Belgium) is mounted on a microscope stand (Leica Microsystems, Wetzlar, Germany) to reduce mechanical vibrations and to allow for a precise positioning. The InSb array (320 × 256 pixels) records infrared radiation between 3 and 5 μm and with a full frame rate $f_{\mathrm{cam}}$ of up to 250 Hz. The camera is calibrated by the supplier and provides temperature images. A PCI Camera-Link-based frame grabber card (PCIe-1427, National Instruments, Austin, TX, USA) is used to transfer the temperature images in real time to a personal computer and to generate synchronization triggers which are sent to the thermal camera and to the alternating magnetic field power supply, respectively. The personal computer sets the stimulation frequency via the frame grabber card, while both the thermal camera and the magnetic field power supply are working in slave mode.

The setup described above is using a quantum detector-based thermal camera (Onca-MWIR-InSb-320, XenICs, Leuven, Belgium). Quantum detectors transform absorbed photon energy directly into released electrons. Even if quantum-based cameras exhibit superior performance in terms of minimal measurable temperature difference, spatial resolution and in particular speed, their cost is still prohibitively high for many applications. Thermal detectors convert the photon energy into heat causing changes in the electrical resistance of a bolometer. They demonstrate a lower sensitivity than quantum-based cameras but they are much more affordable and therefore widely used in industry [28,29]. Part B of Figure 1 schematically presents an alternative measurement setup based on an affordable 288 × 382 pixels microbolometer camera (PI-460, Optris, Berlin, Germany) operating in the 7.5 to 13 μm range. Temperature images are transmitted in real time via USB without the need for a specific frame grabber. Therefore, a single board computer (Raspberry Pi, Raspberry Pi Foundation, Cambridge, UK) can be chosen whereas the general user interface is accessed wirelessly and platform-independently via a web server. A standard Digital-to-Analog card (USB-6008, National Instruments, Austin, TX, USA) is used to generate the stimulation trigger. Microbolometer cameras are functioning in the rolling frame mode meaning that the image acquisition cannot be triggered. For this reason, in this alternative setup, the camera acts as the master clock synchronizing the stimulation source.

In LIT the signal to noise ratio per stimulation cycle is called the sensitivity and is given by

(2) $$S_{e}=2\sqrt{\frac{f_{0}}{Nf_{\mathrm{cam}}}}\times \left(\mathrm{NETD}\right),$$

where $f_{0}$ is the stimulation frequency, $f_{\mathrm{cam}}$ the camera framerate and *N* the number of stimulation cycles. NETD is the Noise Equivalent Temperature Difference of the camera and corresponds to the minimal measurable temperature difference. The InSb sensor has a NETD of 17 mK for a full frame rate of approximately 250 Hz whereas the microbolometer exhibits a full frame rate of 80 Hz for a NETD of 40 mK [30,31]. Both sensors have a similar number of pixels. As a result, based on Equation (2), we have to measure 17.3 times longer using the microbolometer camera-based setup to achieve the same sensitivity as with the setup using the InSb sensor. Nonetheless, the performance of the affordable alternative is sufficient for many applications and when taking into account all the components, the setup is more affordable by approximately one order of magnitude.

### 2.2. Mathematical Modeling of the Sample Holder

**Figure 2:**
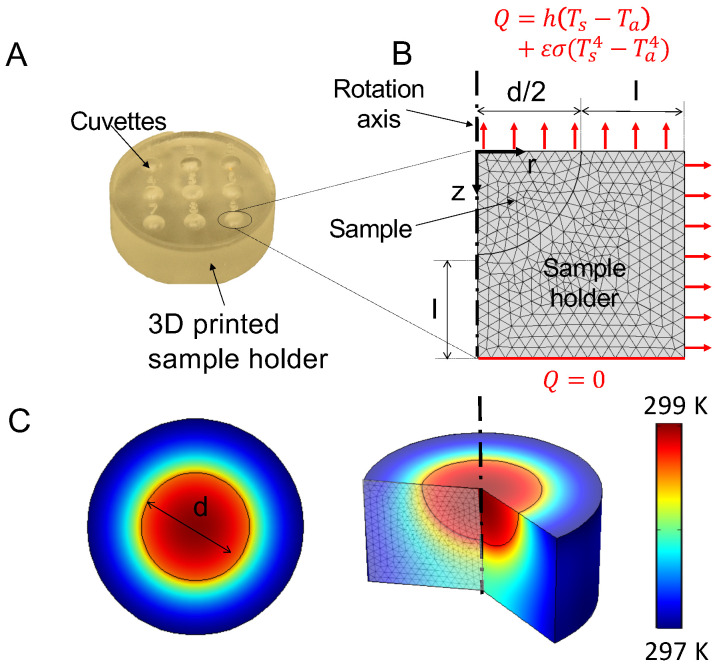
(**A**): Picture of the custom-made polystyrene sample holder designed to investigate NP samples in the liquid state. 9 identical semi-spherical cuvettes allow for the simultaneous investigation of multiple samples. (**B**): 2D axi-symmetrical model of one single cuvette with the surrounding sample holder. The cuvette diameter *d* as well as the sample holder thickness *l* can be varied. The sample is considered to be a homogeneous heat source. Convection and radiation losses take place at the sample and sample holder surfaces, whereas the bottom of the sample holder is insulated. A tetrahedral finite element mesh is used, with a mesh size chosen to ensure numerical stability. (**C**): Simulation results of the sample holder surface and bulk temperature computed using the numerical model described in Section 2.2.

To measure temperature changes in magnetic NPs in liquid samples, a specific sample holder has been designed comprising nine identical half-spherical cavities or cuvettes (See part A of Figure 2). The main goal of the sample holder is to allow for the extraction of quantitative values from the images resulting from the lock-in thermal imaging demodulation and therefore, the dimensions of the cuvettes and of the sample holder need to be carefully chosen [22]. Each cuvette has a radius d/2 whereas the sample holder thickness is set to *l*, which is also the separation distance between two neighboring cuvettes.

In order to investigate the proposed LIT setup in detail, particularly the influence of important parameters such as the sample size, heating slope or stimulation frequency without requiring numerous experimental measurements, we implemented a numerical model of the sample and the sample holder in COMSOL Multiphysics 5.3. To save computational time, a 2D axi-symmetrization was chosen to model a single cuvette (Parts B and C of Figure 2). The sample is considered as a homogeneous half-spherical transient heat source with radius d/2. The time-dependent power generated by one cuvette takes the form

(3) $$P\left(t\right)=\frac{P_{0}}{2}\left(1+\sin\left(\omega_{0}t\right)\right),$$

where $P_{0}$ is the peak power density and $\omega_{0}$ the pulsation where $\omega_{0}=2\pi f_{0}$.

The sample holder is insulated on the bottom side (Q=0) whereas convection and radiation losses take place on the top and on the side of the sample holder including the sample surface (See part B of Figure 2). The heat flux is given by

(4) $$Q=h\left(T_{s}-T_{a}\right)+\sigma \varepsilon \left(T_{s}^{4}-T_{a}^{4}\right),$$

where *h* denotes the heat transfer coefficient, σ the Stefan-Boltzmann constant, ε the surface emissivity, $T_{s}$ the surface temperature and $T_{a}$ the ambient temperature. The sample holder is made of polystyrene ($\rho_{h}$ = 1.05 kg/m^3^, $c_{h}$ = 1.3 J/(kg · K), $k_{h}$ = 0.16 W/(m · K)). The emissivities of the sample surface and the sample holder are set to 0.95 and 0.9, respectively (see Part B Figure 2). The ambient temperature is kept constant at 293.15 K. Initial sample and holder temperature are both set to the ambient temperature $T_{a}$. The heat capacity of the sample *c* is kept constant at 4.18 J/(kg · K). The convection coefficient *h* due to natural convection can be estimated according to standard semi-empirical equations [21] to 5 W/(m^2^· K).

For all simulations presented in this study, the experiment duration is set to 60 s with a variable time step. The mesh is constituted of tetrahedral elements, with sizes chosen to ensure numerical stability. The heat equation

(5) $$\rho c\frac{\partial T}{\partial t}=k\nabla^{2}T$$

is solved on the meshed domain where ρ, *c* and *k* are the local density, heat capacity and heat conductivity respectively.

Part C of Figure 2 demonstrates an example of the sample and holder temperature distribution computed at a defined time after the start of the thermal stimulation (60 s).

Table 2 presents the parameters that have been varied in this study.

If we assume the total thermal energy losses *L* in the environment by conduction, convection and radiation to be linear with temperature, and the sample to be homogeneous inside the cuvette, then the sample temperature evolution over time T(t) is obtained by solving the ordinary differential equation

(6) $$\frac{dT}{dt}\left(t\right)=\beta \tilde{P}\left(t\right)-\frac{T\left(t\right)-T_{a}}{\gamma },$$

representing the standard energy balance equation where $\tilde{P}\left(t\right)=P\left(t\right)/P_{0}$ is the normalized time-dependent power density generated by the sample. The heating slope β is defined as

(7) $$\beta =\frac{P_{0}}{\rho c},$$

with ρ and *c* being the sample mass density and specific heat capacity respectively. γ is defined as

(8) $$\gamma =\frac{c}{L}.$$

A previous study has demonstrated that in the case of a relatively large cuvette, heterogeneity of the sample can lead to inaccurate heating slope calculation [32]. The validity of the assumption of an homogeneous sample holder depends on the Biot number $B_{i}$ defined as

(9) $$B_{i}=\frac{h}{k}l_{c},$$

where $l_{c}$ is a characteristic length of the sample. In this study, *h* varies between 0.1 and 50 W/(m^2^· K) and the cuvette diameter (that can be considered as the characteristic length of the sample) between 1 and 8 mm. With a heat conductivity of water of 0.6 W/(m · K), the Biot number is not larger than 1 and therefore we can consider the sample as homogeneous.

Equation (6) can be solved using the Laplace transform to compute the time-dependent temperature T(t) upon a harmonic stimulation of the form of Equation (3), which leads to

(10) $$T\left(t\right)=T_{a}+\underset{e(t)}{\underset{︸}{\frac{\beta \gamma }{2}\left(1-\left(1-\frac{\gamma \omega_{0}}{1+\gamma^{2}\omega_{0}^{2}}\right)e^{-t/\gamma }\right)}}+\underset{h(t)}{\underset{︸}{\frac{\beta \gamma }{2}\frac{\sin\left(\omega_{0}t\right)-\gamma \omega_{0}\cos\left(\omega_{0}t\right)}{1+\gamma^{2}\omega_{0}^{2}}}},$$

where $T_{a}$ is the initial and constant ambient temperature. As the sample is considered homogeneous, T(t) represents the averaged temperature over the sample surface. In Equation (10) the functions h(t) and e(t) denote the harmonic and exponential parts, respectively. The initial non-steady temperature represented by e(t) is a well-known problem in LIT referred as initial thermal relaxation [23]. The mean temperature of the sample increases until it reaches a quasi-state where it oscillates around a mean value. Breitenstein developed a method to subtract the initial thermal relaxation [23]. Analytically, this is equivalent to subtracting e(t) from the temperature signal. Demodulating the harmonic part h(t) according to the synchronous narrow two-channels correlation we obtain the in-phase $S^{0^{{}^{\circ}}}$ and in-quadrature signal $S^{90^{{}^{\circ}}}$ [23]

(11) $$S^{0^{{}^{\circ}}}=\frac{\beta \gamma^{2}\omega_{0}}{1+\gamma^{2}\omega_{0}^{2}},\;S^{90^{{}^{\circ}}}=\frac{\beta \gamma }{1+\gamma^{2}\omega_{0}^{2}},$$

from which we can calculate the heating slope β

(12) $$\beta =\omega_{0}\frac{{\left(S^{90^{{}^{\circ}}}\right)}^{2}+{\left(S^{0^{{}^{\circ}}}\right)}^{2}}{S^{0^{{}^{\circ}}}},$$

and estimate γ

(13) $$\gamma =\frac{S^{0^{{}^{\circ}}}}{\omega_{0}S^{90^{{}^{\circ}}}}.$$

The total losses value γ can be extracted independently of the sample emissivity and potential homogeneity of the magnetic field that are affecting identically $S^{90^{{}^{\circ}}}$ and $S^{0^{{}^{\circ}}}$. If the total losses take place on a much longer time scale than the stimulation period, i.e., if $\gamma \gg 1/(2\pi \omega_{0})$ or quasi-adiabatic case, then β simplifies to

(14) $$\beta =2A\omega_{0},$$

where *A* is the demodulated signal amplitude defined as

(15) $$A=\sqrt{{\left(S^{90^{{}^{\circ}}}\right)}^{2}+{\left(S^{0^{{}^{\circ}}}\right)}^{2}}.$$

In addition to its simplicity, the use of Equation (14) to calculate the heating slope β exhibits two advantages. First, it is less affected by noise than Equation (12) (See Section 3.3). Second, as the amplitude is insensitive to phase shifts, β can be calculated even in the case where the camera is not synchronized with the stimulation signal. Therefore, Equation (14) is more adapted to the affordable setup proposed in Section 2.1 using a rolling frame microbolometer.

## 3. Results

We present the results of the simulation investigating the cuvette size, the sample holder dimensions, heat losses, stimulation frequency and heating slope using the numerical and mathematical models presented in Section 2.2. The temperature is averaged over the sample surface and the resulting transient signal is exported from COMSOL Multiphysics 5.3 and demodulated according to the synchronous narrow two-channels correlation using a Mathematica script (Wolfram Research, Inc., Mathematica, Version 12.0). The LIT demodulation is achieved either by compensating for the non-steady state initial heating using or without any correction (labeled as ‘Correction’ or ‘No correction’ in the Figures). The heating slope β is computed using Equation (12) or Equation (14) (labeled respectively as ‘$S^{0^{{}^{\circ}}}$ & $S^{90^{{}^{\circ}}}$ ’ or ‘A’ in the Figures).

### 3.1. Sample and Sample Holder Dimensions

We previously reported [22] that the cuvette dimension must fulfil the extended heat source condition, which means that the cuvette diameter *d* (See part B of Figure 2) should be much larger than the thermal diffusion length of the sample defined as

(16) $$\Lambda =\sqrt{\frac{2\alpha }{\omega_{0}}},$$

where α=k/ρc is the thermal diffusivity. For pure water at 20 °C, Λ is approximately 0.2 mm for a stimulation frequency of 1 Hz [33]. A cuvette with a diameter d=4.0mm has been designed which is about 20 times the thermal diffusion length.

**Figure 3:**
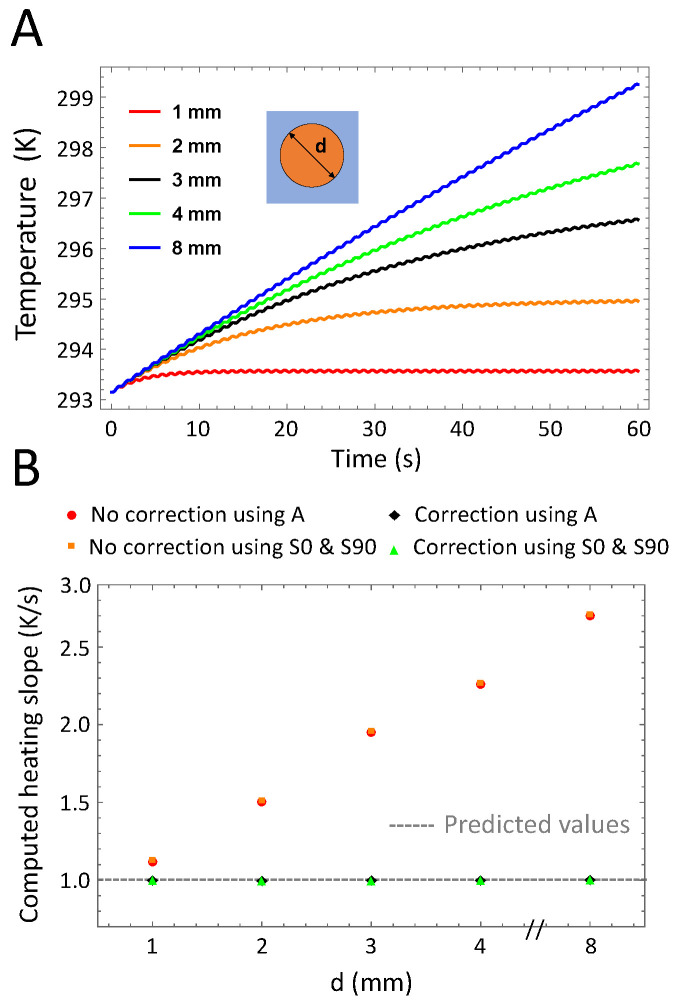
Variations of the cuvette diameter. (**A**): Time-dependent temperature of the sample for different cuvette diameters. For smaller diameters the signal reaches the quasi-steady state faster than for a larger cuvette, for which the signal is still in the thermal relaxation phase after 60 s. (**B**): Computed heating slope for different cuvette diameters. Heating slope values are normalized. As expected, for large cuvettes, a correction of the non-initial heating is required to extract accurate values. SAR calculations using the in-phase and in-quadrature signal or the amplitude signal alone lead to similar values.

Part A of Figure 3 presents the time-dependent temperature averaged over the whole cuvette area for different cuvette diameters ranging from 1 to 8 mm. Parameters other than *d* are kept constant in these simulations (*l* = 2 mm, $f_{0}$ = 1 Hz, *h* = 5 W·m^−2^·K^−1^ and $P_{0}$ = 5.05 × 10^5^ W·m^−3^). If the signal amplitude is unaffected by the size of the cuvette, the larger the area the longer it takes for the temperature to reach quasi-equilibrium. Therefore, a correction for the thermal relaxation time is particularly required for large cuvette sizes. The time-dependent temperature is demodulated to compute the heating slope β, which is proportional to the peak power density. Normalization of β is achieved by dividing it by the target value set in the numerical model. As demonstrated in Part B of Figure 3, if the thermal relaxation phase is taken into account, β can be retrieved accurately independently of the cuvette size. The extended heat source condition is therefore not required, as reported previously [21], if the temperature signal is spatially averaged over the cuvette surface area.

In addition to the sample size, the dimensions of the sample holder should also be carefully selected. Previously, we set the sample holder thickness *l* much larger than Λ to ensure that the sample can be considered thermally thick [21]. This implies that we also applied the same rule to the minimal distance separating two neighboring cuvettes to mitigate the thermal interaction between the samples. In order to build a thermally thick sample holder, we chose polystyrene, which demonstrate a thermal diffusion length λ=0.19mm at a stimulation frequency of 1 Hz [33]. The sample holder thickness was set to be at least ten times larger. Therefore, the cuvettes were separated by 1.9 mm.

**Figure 4:**
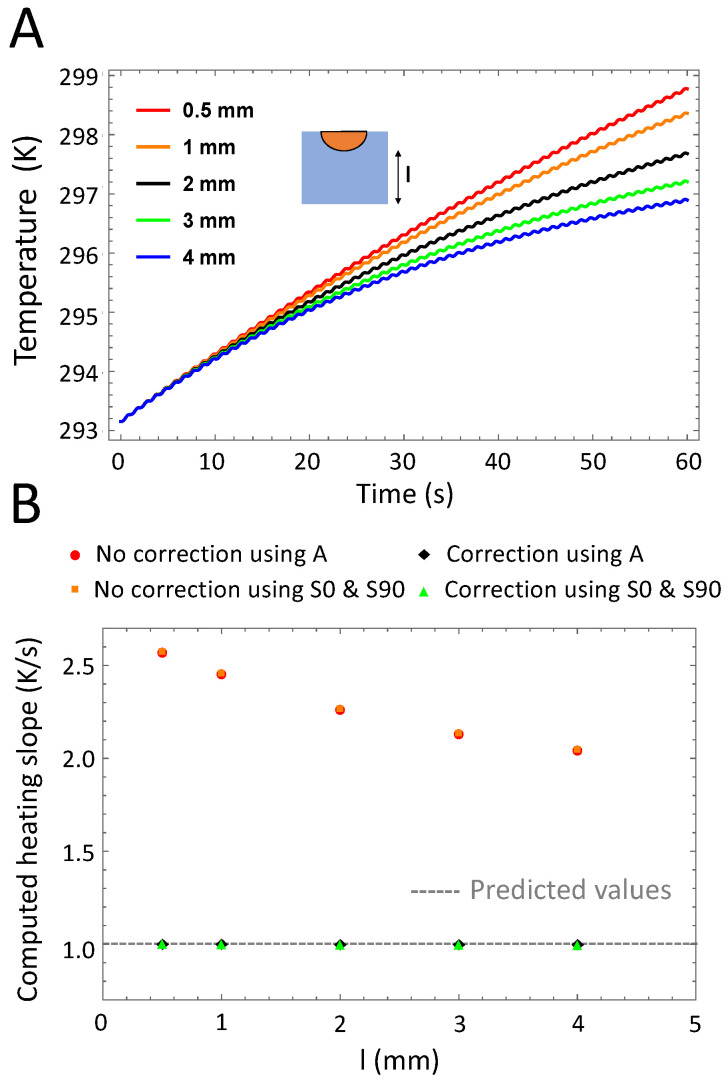
Variations of the sample holder thickness. (**A**): Time-dependent temperature of the sample for different sample holder thickness values *l*. For larger thickness, the signal reaches the quasi-steady state faster than for a thinner sample holder, for which the signal is still in the thermal relaxation phase after 60 s. (**B**): Computed heating slope β (normalized) for different sample holder thickness values. Heating slope calculations using the in-phase and in-quadrature signal or the amplitude signal alone lead to similar values. Correction of the thermal relaxation phase is required, in particular for thinner sample holders.

We calculated the time-dependent temperature averaged over the whole cuvette area for different values of the sample thickness *l* varying from 0.5 to 4 mm (Part A of Figure 4), while all other parameters are kept constant (*d* = 2 mm, $f_{0}$ = 1 Hz, *h* = 5 W·m^−2^· K^−1^ and $P_{0}$ = 5.05 × 10^5^ W·m^−3^). As expected, the signal amplitude remains unaffected by the sample thickness, which still influences the thermal relaxation phase. Nonetheless, β can still be retrieved with high accuracy (Part B of Figure 4) offering much more flexibility in the sample holder design than previously reported [21,22].

### 3.2. Heat Losses and Stimulation Frequency

**Figure 5:**
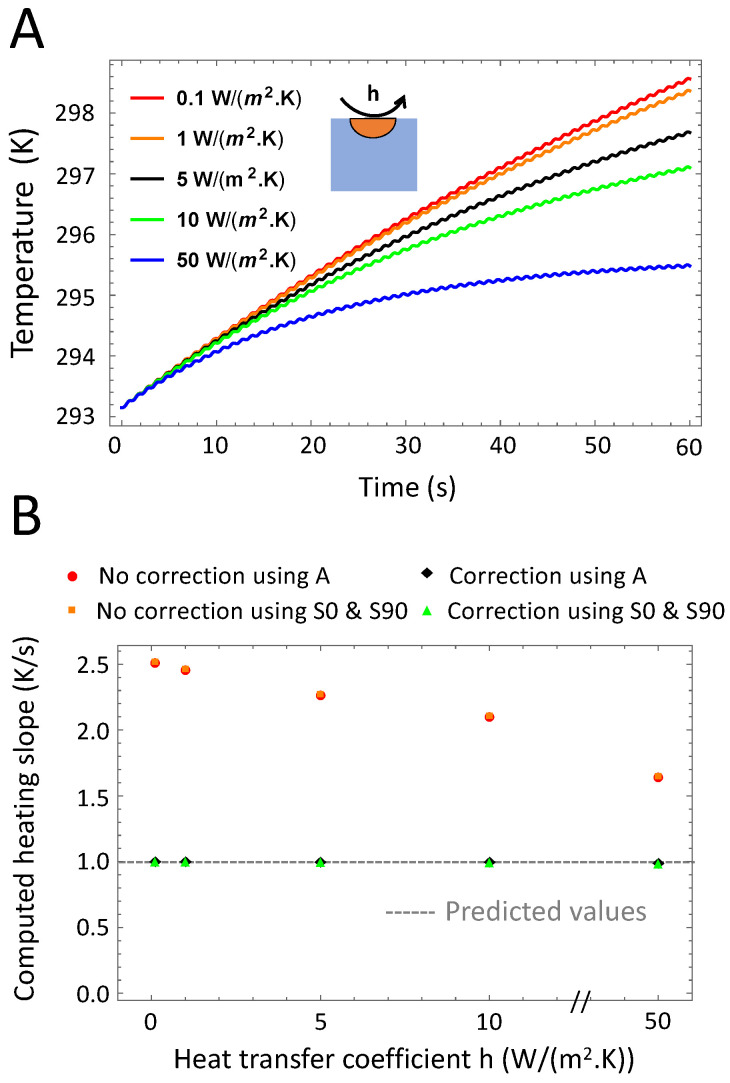
Variations of the heat loss coefficient. (**A**): Time-dependent temperature of the sample for different Newton coefficients *h*. For high *h* values, the thermal relaxation phase is shorter. (**B**): Computed β (normalized) for different values of *h*. Correction for the initial heating is required especially for high *h* values, for which the thermal relaxation phase is longer. β calculations using the in-phase and in-quadrature signal or the amplitude signal alone lead to similar values.

We hypothesized in Section 2.2 that if the total convection, conduction and radiation losses are much larger than the stimulation period, i.e., $\gamma \gg 1/(2\pi \omega_{0})$, the relation between the demodulated signal amplitude *A* and β simplifies to Equation (14). Figure 5 investigates the effect of convection using the convection coefficient *h* as the major contributor to the total (thermal energy) loss in the sample, *L*. The higher *h* the faster the signal reaches quasi-steady state (See part A of Figure 5). Therefore, a compensation is particularly needed for low convection. Nonetheless, we notice from Figure 5B that for *h* varying between 0.1 to 50 W/(m^2^·K) and for a stimulation frequency of 1 Hz we remain in the quasi-adiabatic regime and that the heating slope can be computed with high accuracy using the amplitude only.

**Figure 6:**
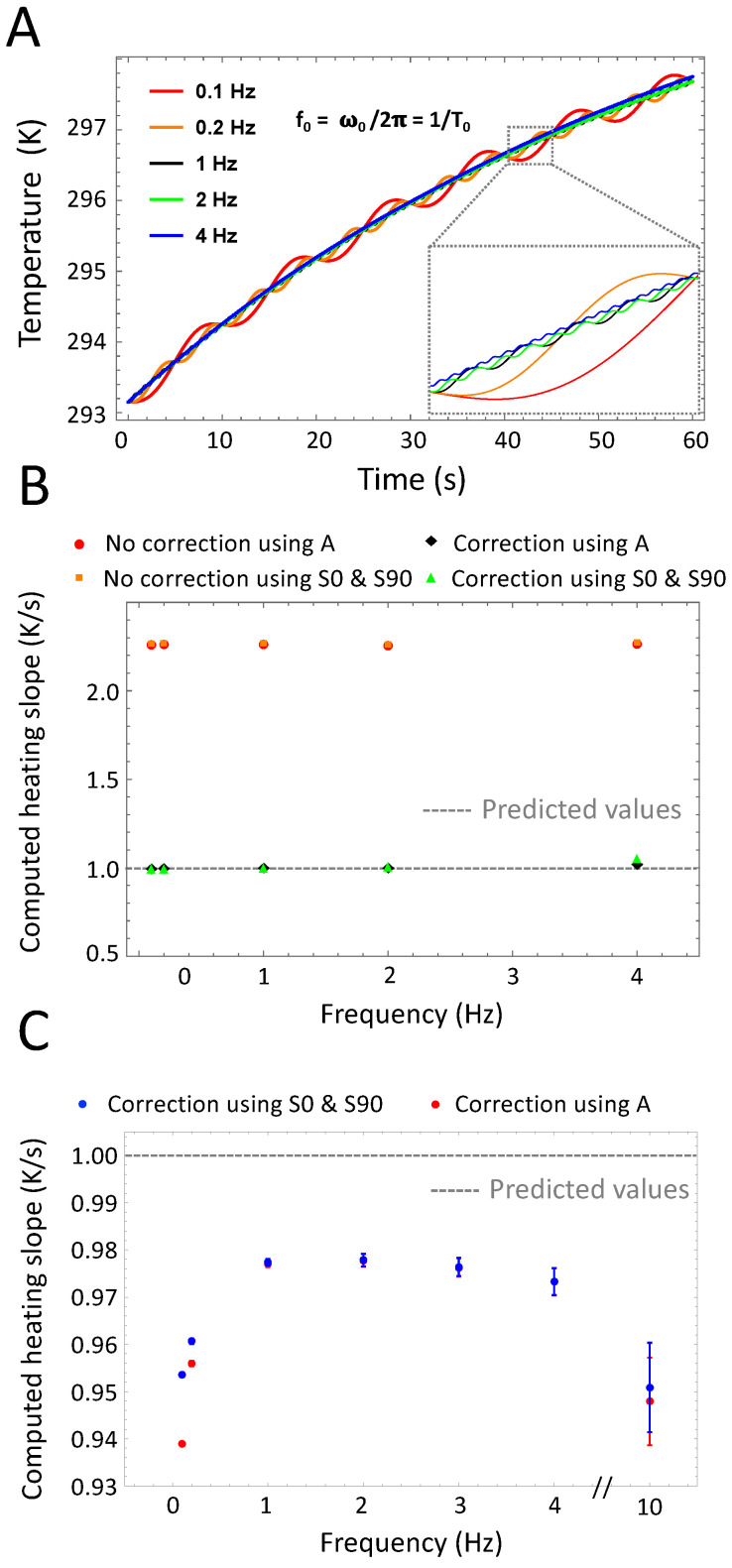
Variations of the stimulation frequency. (**A**): Time-dependent temperature of the sample for different stimulation frequencies. The stimulation frequency exhibits no effect on the duration of the thermal relaxation phase. (**B**): Computed heating slope β (normalized) for different stimulation frequencies. Correction for the unsteady initial heating is required for all frequencies. (**C**): Corrected heating slope values computed using the in-phase and in-quadrature signals or the amplitude alone. At low frequencies (less than 1 Hz), the exact calculation using the in-phase and in-quadrature signals leads to slightly more accurate results.

In LIT experiments, the stimulation frequency $f_{0}$ (also called modulation frequency) is a major experimental parameter. Figure 6 describes the effect of changing the stimulation frequency on the transient surface temperature of the sample and the determination of the heating slope. Changing the frequency from 0.1 Hz to 4 Hz directly influences the signal sinusoidal part h(t) without changing the exponential heating e(t) in Equation (10) (See Part A of Figure 6). Part B of Figure 6 demonstrates the demodulated heating slope obtained using either $S^{90^{{}^{\circ}}}$ and $S^{0^{{}^{\circ}}}$ or using only the amplitude signal. Correction of the relaxation phase is necessary at all stimulation frequencies but if achieved leads to an accurate determination of β. In a second step we investigated more in detail the influence of the stimulation frequency on the setup sensitivity by adding white noise simulating a camera NETD of 40 mK. The experiment and β extraction were repeated *in silico* 100 times to compute the mean and standard deviation of the extracted heating slope (See Part C of Figure 6). As previously reported in the literature, higher frequencies lead to a higher standard deviation due to a lower amplitude signal [23]. On the other hand, lower frequencies below 1 Hz are less accurate and they lead to slightly lower mean values. 1 Hz appears to be a satisfying trade-off.

### 3.3. Heating Power

**Figure 7:**
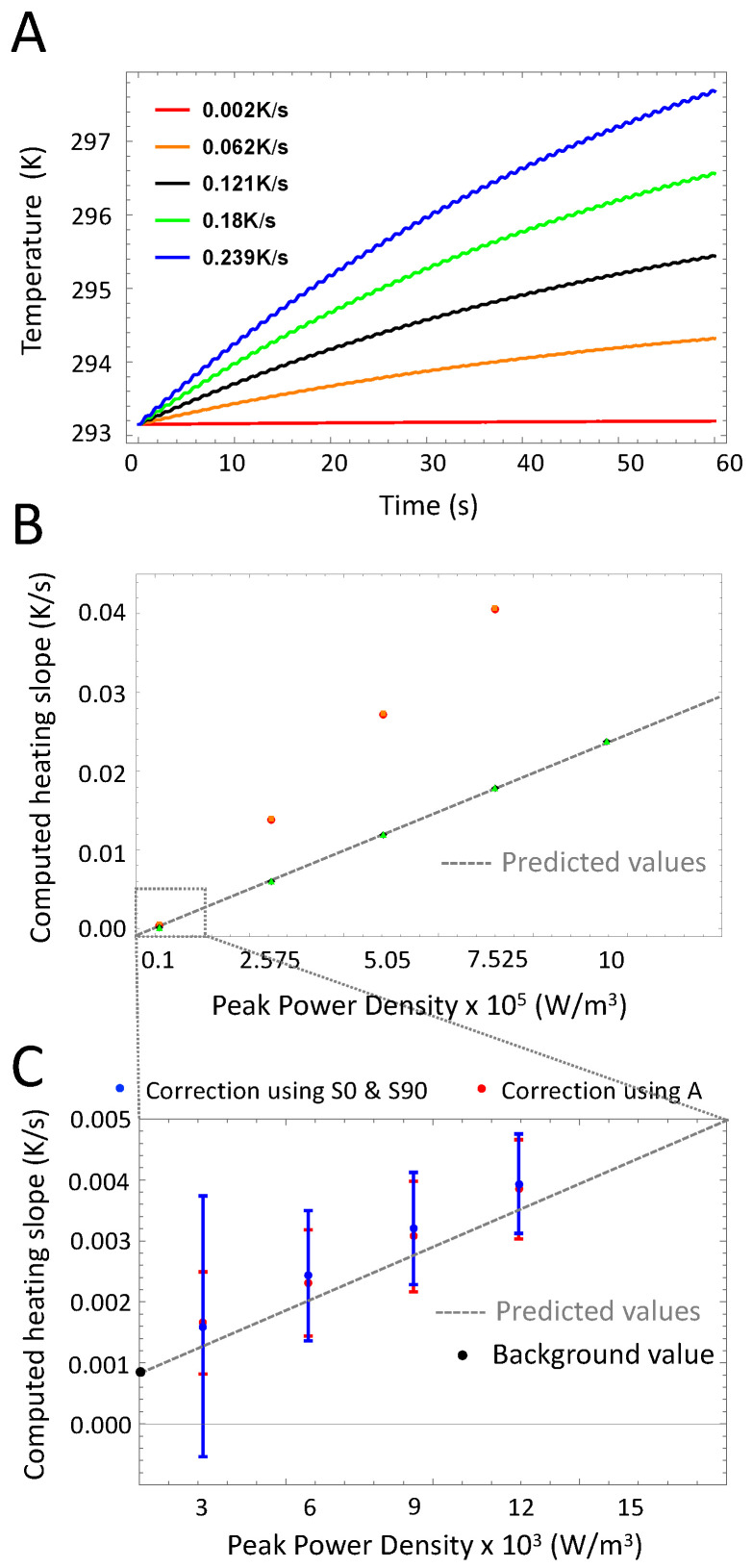
Variations of the heating slope. (**A**): Time-dependent temperature of the sample for different values of the heating slope. The heating slope influences the duration of the thermal relaxation phase as well as the signal amplitude. (**B**): Computed heating slope β (normalized) for different peak power density. Intuitively, the calculation using the amplitude is preferable in terms of sensitivity due to the ratio in Equation (12). (**C**): Extended view of the computed heating slope for low power values. Dots represent mean values whereas error bars the standard deviation, computed for 100 measurements. The black dot represents the experimental value of the background signal measured outside the cuvettes.

LIT is an averaging method, which exhibits very high sensitivity allowing measuring temperature differences in the mK range. Therefore, the technique is particularly well suited to measure highly diluted samples or NPs exhibiting low heating power. Figure 7 investigates the lowest heating slope that can be measured for standard experimental parameters ($f_{0}$ = 1 Hz, *h* = 5 W/(m^2^· K), 60 stimulation cycles). The heating slope β strongly affects the signal amplitude and the thermal relaxation time (See Part A of Figure 7). As a result, a compensation of the initial non-steady phase is important at high heating slope only (See Part B of Figure 7). Experimentally, we previously reported that LIT was able to measure a heating slope as low as 0.87 mK/s [22]. Such values have been obtained using the background level of the amplitude images around the cuvette where no signal is present (Part D of Figure 2). Calculating β more than 100 times and computing the signal’s mean and standard deviation we notice two interesting phenomena. First, at low heating the standard deviation increases dramatically leading to high inaccuracy when computing the heating slope. Second, using the in-phase and in-quadrature signals to extract β leads to higher variability. As a result, it is better to use the amplitude to calculate the heating slope as we previously validated that the quasi-adiabaticity is almost guaranteed.

To determine experimentally the lowest heating slope β that can be measured with our instrument, we prepared SPIONs at different concentrations. Particle batches of 3 different sizes (21 ± 4 nm, 24 ± 6 nm and 25 ± 2 nm) were prepared by thermal decomposition [22]. Samples were diluted to varying concentrations (0.1, 0.2, 0.5 and 1 mg Fe/mL) and loaded into the sample holder (Part A of Figure 2). The SPIONs concentration was determined by weighing a known volume of pre-evaporated SPIONs dispersion. The AFM generator was set to a frequency of 524.5 kHz and magnetic field intensity of 18.5 mT. The other parameters were identical as in the simulation: A stimulation frequency of 1 Hz was chosen, 60 cycles were acquired and the camera frame rate was set to 200 Hz.

**Figure 8:**
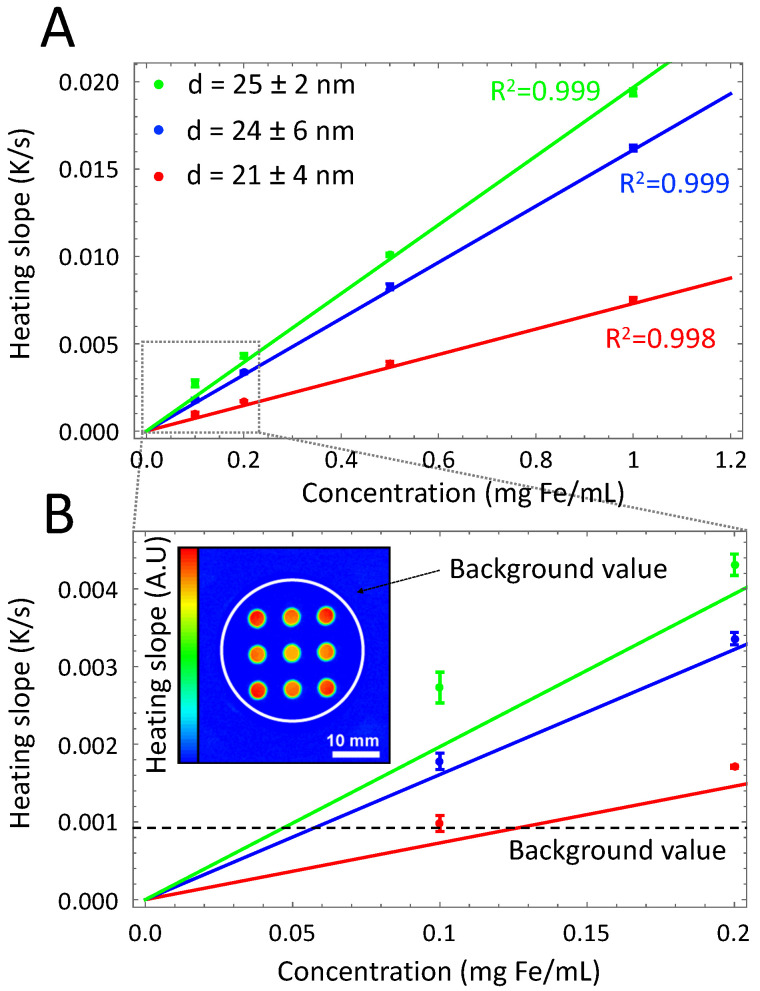
(**A**): Investigation of SPIONs with different size using measurements of the heating slope as a function of magnetic NP concentration for various sizes (triplicate experiment). Solid lines show best fit linear regression curves through the data. (**B**): Lower concentrations, approaching the limit of detection. In agreement with the model, the standard deviation increases at lower heating slopes. We plotted the value of the background that was previously used as limit of detection. Inset: Example of an heating slope image where the nine cuvettes are loaded with an identical sample. The homogeneous background signal outside the cuvette area was previously used to estimate the minimal measurable signal. Data taken with permission from [22].

Part A of Figure 8 represents the heating slope as a function of SPIONs concentration for different sizes obtained using Equation (14) and after correcting for the initial thermal relaxation time (See Section 2.2). Data points represent triplicate experiments (standard deviation shown as error bars), and solid lines the best fit linear regression curves (R^2^ coefficients are displayed in color).

As expected by theory [3,34,35], the heating slope is proportional to the iron concentration. Part B of Figure 8 shows an extended view at lower concentrations. In agreement with simulation results, measurement accuracy is lower with a higher standard deviation. The background signal represents the demodulated signal of the sample holder surface outside the cuvette area and was previously considered as the minimum signal detectable (See Inset in Figure 8) [22]. Simulations performed in this study demonstrated that measurement accuracy decreases in a non negligible manner at lower signal intensity (See part C of Figure 7) and that the background signal does not represent a valid estimation of the minimal measurable signal.

**Figure 9:**
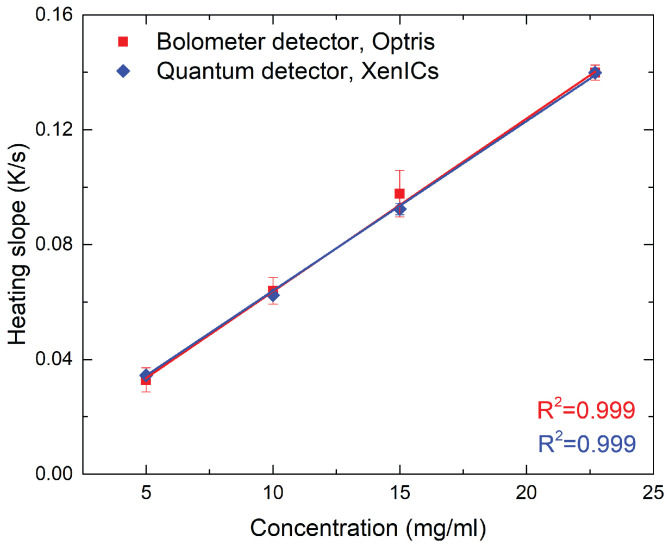
Comparison of LIT thermal investigations on 21 nm SPIONs using a microbolometer sensor (PI-460, Optris, Berlin, Germany) and a quantum sensor (Onca-MWIR-InSb-320, XenICs, Leuven, Belgium) (triplicate experiment). Solid lines show best fit linear regression curves through the data. To compensate for the lower sensitivity of the microbolometer sensor, measurement times were adjusted, i.e., 30 s and 500 s were set for the Onca camera and the PI-460, respectively.

Finally, to assess the performance of the affordable apparatus proposed in this study, 21 nm SPIONs were measured with both setups (see Figure 9). To account for the lower sensitivity of the microbolometer sensor, the measurement time was roughly increased by a factor 17. The heating slopes are proportional with the iron concentration and both devices deliver almost identical values.

## 4. Conclusions

In this study we demonstrated a lock-in thermal imaging setup specifically designed to investigate temperature changes in liquid samples containing stimuli-responsive magnetic NPs. More specifically, we recapitulate the measurement principle and apparatus, and proposed and experimentally tested a affordable alternative, which could help to facilitate the application of LIT in industry. Simulations were performed and compared to experimental results, which have been published by previous studies. In detail, we investigated key parameters for lock-in thermal imaging, such as sensitivity, thermal diffusion length, losses, stimulation frequency and heating slope. Furthermore, we modeled the influence of the sample holder on the heating processes in order to define its optimal dimensions. Our findings have shown that LIT is an excellent tool for the thermal analysis of magnetic NPs liquid samples, as it accurately resolves slight temperature differences (i.e., high sensitivity), prevents lateral heat spreading and thereby reduces thermal losses to the environment. In terms of stimulation frequency we found 1 Hz to be the best trade-off between accuracy and amplitude signal. Additionally, our simulations showed that cuvettes with a diameter of 1 mm exhibit the most accurate values, while a sample holder with a large thickness enhances the thermal relaxation of the system.

## Figures and Tables

**Table 1:** Overview of common methods used for the characterisation of nanomaterial heat generation.

	Method			
**Parameter**	**LIT**	**ST**	**FOC**	**TC**
Sensitivity	high	low	high	high
Invasive	no	no	yes	yes
Sample system	solid & liquid	solid & liquid	liquid	liquid
Field of view	large	large	narrow	narrow

**Table 2:** The cuvette diameter *d* (Figure 1), the sample holder thickness *l* (Figure 1), the stimulation frequency $f_{0}$, the convection loss *h* and the peak power density $P_{0}$, proportional to the heating slope β, have been varied in this parametric study.

*d* [mm]	*l* [mm]	$f_{0}$ [Hz]	*h* [W/(m^2^ · K)]	$P_{0}$ [W/m^3^]
1	0.5	0.1	0.1	10^4^
2	1	0.2	1	2.575 × 10^5^
3	2	1	5	5.05 × 10^5^
4	3	2	10	7.525 × 10^5^
8	4	4	50	10^6^
